# Estimation of Bedtimes of Reddit Users: Integrated Analysis of Time Stamps and Surveys

**DOI:** 10.2196/38112

**Published:** 2023-01-17

**Authors:** William U Meyerson, Sarah K Fineberg, Ye Kyung Song, Adam Faber, Garrett Ash, Fernanda C Andrade, Philip Corlett, Mark B Gerstein, Rick H Hoyle

**Affiliations:** 1 Department of Psychiatry & Behavioral Sciences Duke University School of Medicine Durham, NC United States; 2 Department of Molecular Biochemistry & Biophysics Yale University New Haven, CT United States; 3 Program in Computational Biology & Bioinformatics Yale University New Haven, CT United States; 4 Department of Psychiatry Yale University New Haven, CT United States; 5 Durham Veterans Affairs Healthcare System Durham, NC United States; 6 Center for Medical Informatics Yale University New Haven, CT United States; 7 Veterans Affairs Connecticut Healthcare System West Haven, CT United States; 8 Department of Psychology and Neuroscience Duke University Durham, NC United States; 9 Wu Tsai Institute Yale University New Haven, CT United States; 10 Department of Computer Science Yale University New Haven, CT United States; 11 Department of Statistics & Data Science Yale University New Haven, CT United States

**Keywords:** social media, sleep, parametric models, Reddit, observational model, research tool, sleep patterns, usage data, model, bedtime

## Abstract

**Background:**

Individuals with later bedtimes have an increased risk of difficulties with mood and substances. To investigate the causes and consequences of late bedtimes and other sleep patterns, researchers are exploring social media as a data source. Pioneering studies inferred sleep patterns directly from social media data. While innovative, these efforts are variously unscalable, context dependent, confined to specific sleep parameters, or rest on untested assumptions, and none of the reviewed studies apply to the popular Reddit platform or release software to the research community.

**Objective:**

This study builds on this prior work. We estimate the bedtimes of Reddit users from the times tamps of their posts, test inference validity against survey data, and release our model as an R package (The R Foundation).

**Methods:**

We included 159 sufficiently active Reddit users with known time zones and known, nonanomalous bedtimes, together with the time stamps of their 2.1 million posts. The model’s form was chosen by visualizing the aggregate distribution of the timing of users’ posts relative to their reported bedtimes. The chosen model represents a user’s frequency of Reddit posting by time of day, with a flat portion before bedtime and a quadratic depletion that begins near the user’s bedtime, with parameters fitted to the data. This model estimates the bedtimes of individual Reddit users from the time stamps of their posts. Model performance is assessed through k-fold cross-validation. We then apply the model to estimate the bedtimes of 51,372 sufficiently active, nonbot Reddit users with known time zones from the time stamps of their 140 million posts.

**Results:**

The Pearson correlation between expected and observed Reddit posting frequencies in our model was 0.997 on aggregate data. On average, posting starts declining 45 minutes before bedtime, reaches a nadir 4.75 hours after bedtime that is 87% lower than the daytime rate, and returns to baseline 10.25 hours after bedtime. The Pearson correlation between inferred and reported bedtimes for individual users was 0.61 (*P*<.001). In 90 of 159 cases (56.6%), our estimate was within 1 hour of the reported bedtime; 128 cases (80.5%) were within 2 hours. There was equivalent accuracy in hold-out sets versus training sets of k-fold cross-validation, arguing against overfitting. The model was more accurate than a random forest approach.

**Conclusions:**

We uncovered a simple, reproducible relationship between Reddit users’ reported bedtimes and the time of day when high daytime posting rates transition to low nighttime posting rates. We captured this relationship in a model that estimates users’ bedtimes from the time stamps of their posts. Limitations include applicability only to users who post frequently, the requirement for time zone data, and limits on generalizability. Nonetheless, it is a step forward for inferring the sleep parameters of social media users passively at scale. Our model and precomputed estimated bedtimes of 50,000 Reddit users are freely available.

## Introduction

Adequate sleep is vital for health and well-being, and the timing of when we sleep matters too [1-4]. With social and environmental pressures to awake early, individuals with later bedtimes suffer either from sleep loss or from absenteeism and other social consequences of late rising [5]. Teens with later bedtimes than their parents have more unsupervised hours with which to associate with like peers to pursue risky activities. Whatever the cause, multiple studies demonstrate that individuals with later bedtimes are at increased risk of mood disorders and substance use disorders [6,7]. Thus, bedtimes are emerging as an important health metric alongside other sleep-related parameters.

To generate hypotheses about the causes and consequences of late bedtimes and other sleep features, some researchers are turning to social media as a data source. If researchers had access to information about the sleep patterns of social media users, then they could test those sleep patterns for associations with any of the limitless web-based and offline behaviors of those users recorded in their social media activity. In general, researchers do not know the sleep patterns of social media users unless they recruit them for a study; unfortunately, this means that whatever advantages social media data otherwise affords in terms of scale and passive monitoring are lost to sleep research. A special use case of these data would be for understanding the role of social media use in affecting sleep patterns, which is a topic of significant interest [8-14]. A recent systematic review concluded that the sleep and social media literature is limited by its reliance on surveys and that for the field to move forward, new study designs are needed [15].

A number of pioneering studies have attempted to infer sleep patterns directly from social media data. These attempts have used 3 strategies: manual coding, linguistic mining, and time stamp analysis. One qualitative study used manual coding to identify themes of sleep difficulties in a subset of 192 tweets of pregnant mothers during the COVID-19 pandemic [16]. A linguistic study inferred the presence of insomnia among Twitter users from insomnia-related keywords such as “can’t sleep” and showed that users with these key phrases post more nighttime tweets [17]. Another study using linguistic mining identified Twitter users with insomnia based on public self-reports of insomnia and then trained a model to identify insomnia using the linguistic features of these users [18]. Another study calculated what they term as the “pseudo-sleeping time” from the difference in time stamps between tweets that effectively say “good morning” and preceding tweets that effectively say “good night” [19].

While these early attempts to infer sleep patterns from social media data are innovative, they are subject to a number of limitations that affect their credibility or scope. What the field requires is a suite of approaches that (1) cover the range of sleep parameters of interest, (2) demonstrate the validity of their inferences, (3) are robust across contexts, (4) are scalable, (5) cover the range of social media platforms of interest, and (6) come with software to allow for dissemination and improvement. The manual coding required in Talbot et al [16] is not scalable. Detection of linguistic features associated with insomnia as in McIver et al [17] and Sakib et al [18] has its uses but is a different sort of goal than our goal of inferring sleep parameters for a more general study of sleep. Moreover, linguistic features are context dependent, so it is unclear how they will replicate across contexts [20]. The time stamp study of [19] did not empirically test whether the quantity they calculate of “pseudo-sleeping time” is a valid proxy for a quantity we care about (such as sleep duration). All of the profiled studies apply to Twitter data, which is just 1 platform. None of these papers came with associated software that other researchers can use.

This study builds on these pioneering studies to address some of their limitations. We estimated the bedtimes of Reddit users from the time stamps of their posts, tested the validity of these inferences against survey data, and released our model as an R package. Our work adds a sleep parameter of interest (estimated bedtime) to those that are covered by some members of the suite of available approaches. We demonstrate the validity of our inferences using survey data. We believe that grounding our method in time stamps—objective, machine-readable partial listings of times when users are awake—makes the method intrinsically more likely to be robust across contexts than are linguistic features. We demonstrate the scalability of our approach by estimating the bedtimes of 50,000 users. Because our model is designed for the social platform Reddit whereas other profiled models are for Twitter, our tool increases the number of platforms that are served by the suite of approaches. The R package released alongside this paper allows other researchers to reproduce, apply, and extend our work.

## Methods

### Overview of Approach

This section gives a general overview of the model and pipeline; implementation details are described in subsequent sections.

We obtained the time stamps of the Reddit posts of 159 eligible Reddit users who publicly reported or privately reported on our survey both their average bedtime and their time zone of residence. These time stamps and reported bedtimes were used to train a simple parametric model that estimates bedtimes from the time stamps of users’ posts. To a first approximation, the model can be described as looking for the hour of the night at which a user’s average posting rate across nights typically starts falling.

In general, we cannot infer the bedtime of a user just from when he or she stops posting to Reddit on a given night, because the user may simply be awake but otherwise occupied. Instead, we look for the general trend across nights of the hour of the night when the user is less likely to post than before. When we average across nights for a user, we look for a decrease in the rate of posting rather than a cessation of posting because of variation in a user’s bedtime from night to night.

Since the model has few parameters, the main test of the model is its goodness of fit to the training data. As an additional reassurance against overfitting, we employ k-fold cross-validation in which all 159 eligible labeled users are included in one run or another in the training set and in the testing set. The final model uses all labeled data in the training. The model is then applied to the application set, the remaining 50,000 users for whom time zone data but not bedtime data are available.

### Data Acquisition and Initial Processing

#### Producing Private Data

For model training and testing, we required information about Reddit users regarding their average bedtime and time zone of residence. A Reddit ad was placed asking adult volunteers to complete a Qualtrics survey (Qualtrics International Inc) regarding their Reddit username, typical bedtime, range in bedtime, country, and state of residence. In the survey, the participants were asked to select their typical bedtime from a drop-down menu with a resolution of 30 minutes.

#### Extracting Publicly Self-reported Bedtimes and Time Zones

In soliciting participants for our survey, we encountered insufficient sample size. Therefore, additional self-reported bedtimes and time zones from the universe of public posts of Reddit users were obtained.

Preliminary investigation of public forums on Reddit revealed that users from time to time post a public message asking members of a Reddit community about their bedtime habits, such as (hypothetically) a post in a gardening-related forum asking “Fellow gardeners of Reddit, when do you go to bed?” to which other Reddit users reply publicly in the comments with responses such as “I usually go to bed at 8 pm.” To systematically extract these informal preexisting public polls, the Reddit API wrapper for python, PRAW (python version 3.7, Python Software Foundation) was used in August 2021 to obtain a set of search results representing candidate existing public surveys of sleep habits on Reddit, with search queries “when do you”+“bed,” “when do you”+“sleep,” “what time do you”+“sleep,” “when is your bedtime,” “what is your bedtime,” and “what is your bed time,” yielding 1570 candidate public surveys posts in which Reddit users informally ask each other when they go to bed. YKS screened these public surveys for relevance, identifying 353 relevant surveys. We next obtained the public responses to these public surveys using another PRAW query; a total of 5357 responses to these surveys were obtained. YS, AF, and WM coded each free-text response to HH:MM AM/PM format according to the coding rules in Multimedia Appendix 1, which yielded 2445 responses that could be interpreted as a single numeric bedtime. For 319 users who self-reported bedtimes in more than one survey, we calculated the circular mean bedtime with the CircStats R package (R version 3.5), yielding 2126 unique users for whom we have publicly self-reported bedtimes. For downstream analyses, publicly reported bedtimes were rounded to the nearest 15-minute interval.

Because Reddit reports time stamps in universal coordinated time (UTC) while users presumably announce their bedtimes in their local times, the next step was to identify users who publicly reveal their location and from this deduce their time zone. The smgeo Python package for geolocating Reddit users includes in its training data more than 50,000 Reddit users who have publicly self-reported their locations, and we obtained access to these users and their locations by completing a data usage agreement [21]. From these locations, we inferred time zones using the lutz R package. Of these users, 128 had publicly reported bedtime data identified as above.

#### Downloading Time Stamps

All users’ posts on Reddit from August 2005 to June 2021 were downloaded from the PushShift database in accordance with Reddit’s terms of service [22]. Time stamps were extracted with the jq command line tool.

#### Bot Filtration

Bots represent a significant portion of traffic on social media, and attention must be paid to reducing their influence on study results [23]. We considered the prompt-appropriate responses of users who publicly or privately reported their bedtimes to be sufficient evidence that those users were not bots. For the application data set, we flagged users as suspected bots based on chosen parameters for suspicious volume speed (maximum posts in a minute were ≥9), volume (total posts in the decade ≥ 2^14^), and timing (≥2% of all posts occurring the same exact minute each day) of posting activity, or if the username contained the substring “bot,” “admin,” “mod,” or “auto.”

#### Inclusion and Exclusion Criteria

For model training, passive research subjects were included if all 3 of the following inclusion criteria were met and the exclusion criterion was not met. The inclusion criteria were as follows: (1) the user’s publicly self-reported bedtime was discoverable and interpretable by our search strategy and coding rules; (2) the location of the user was coded by the training data from [21] with enough precision to identify the user’s time zone; and (3) the user had contributed at least 250 lifetime nondeleted Reddit posts by June 2022. The exclusion criterion was as follows: the user’s reported bedtime was more than 2 SDs above or below the mean reported bedtime of all otherwise-included members of the training set.

For model training, recruited research participants were included if all of the following inclusion criteria were met and the exclusion criterion was not met. The inclusion criteria were as follows: (1) the user consented to participate and completed the required fields of the survey (username, single numerical typical bedtime, and location); (2) the user was 18 years or older to satisfy institutional review board (IRB) requirements; and (3) the user had contributed at least 250 lifetime nondeleted Reddit posts by June 2022. The exclusion criterion was as follows: the user’s reported bedtime was more than 2 SDs above or below the mean reported bedtime of all otherwise-included members of the training set.

For model testing, all participants who met the training criteria indicated above were included.

For posttraining model application, passive research subjects were included if both of the following inclusion criteria were met and the exclusion criterion was not met. The inclusion were as follows: (1) the location of the user was coded by the training data from Harrigian [21] with enough precision to identify the user’s time zone; (2) the user had contributed at least 250 lifetime nondeleted Reddit posts by June 2021 (1 year earlier than the smaller training and testing sets; because of the size of the application set, it was neither practical nor necessary to update the included time stamps after the project start). The exclusion criterion was as follows: the user was flagged as a bot (see the *Bot Filtration* section; this exclusion criterion was not applied to the training or testing data because the prompt-appropriate responses of those users were taken as evidence of human activity).

### Model Development

**Figure 1:**
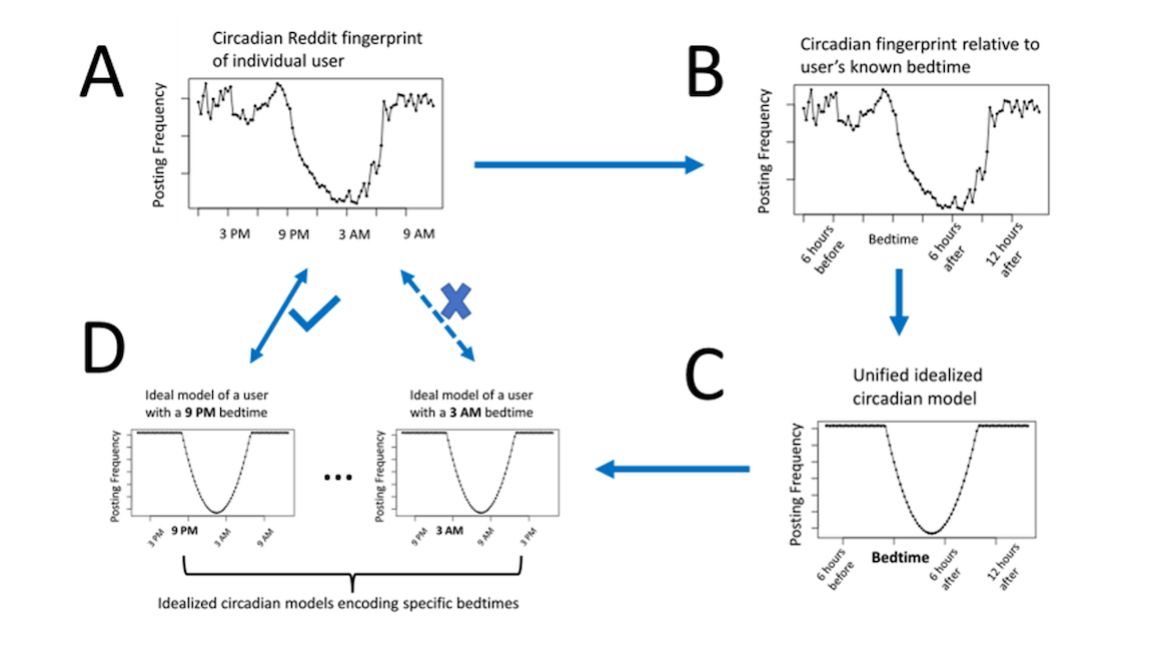
Model schematic. (A) The circadian fingerprint of each Reddit user is calculated by computing the percentage of a user’s Reddit posts occurring at each time of day. (B) The circadian fingerprint of each user is recast in terms of hours relative to the reported bedtime of the user. (C) A unified idealized circadian model is fit against the bedtime-relative fingerprints across many users. (D) By recasting the bedtime-point of a unified idealized model as various clock times, a series of bedtime-specific idealized models are constructed. In model application (arrows connecting D and A), a user’s bedtime is estimated as the bedtime encoded by the bedtime-specific idealized model that best fits the user’s actual circadian fingerprint.

For each Reddit user in the training data, we tabulated a circadian fingerprint that summarizes how frequently that user posts to Reddit at one time of the day versus another (Figure 1A). A user’s circadian fingerprint is a vector of length 96 with each element corresponding to the percentage of the users’ posts across dates that were posted within each 15-minute interval of the 24-hour day. Typically, these fingerprints show a drop in posting frequency in the nighttime hours. We hypothesized that this decreased nighttime posting represents the usual sleeping times of the users. To test this hypothesis, we related users’ reported bedtimes to features of those users’ circadian fingerprints. Individual fingerprints are subject to noise, which we addressed by combining fingerprints from different users. However, straightforward averaging or adding fingerprints from different users would result in dilution of signal because different users have different bedtimes. By recasting users’ circadian fingerprints in a coordinate system zeroed on the users’ respective bedtime (Figure 1B), we were able to combine users’ fingerprints without dilution of a signal. The unified model was then fit to the curve of the combined bedtime-relative fingerprints (Figure 1C). The unified model was chosen on visual inspection to be piecewise quadratic, with a parabola fit to the nighttime lull in Reddit posting activity, bounded on either side with a flat horizontal line with a height that makes the total posting frequency sum to 1. The cut points of transition from a flat line to parabola and back again to the flat line were optimized by considering all possible cut points and choosing the ones with the lowest mean squared error.

More formally, the fraction *r* of a user’s total Reddit posts falling within a given 15-minute interval of the 24-hour day is modeled as:



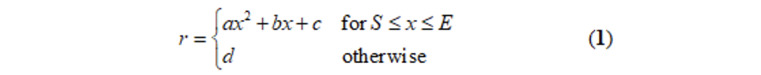



where *x* is the number of hours after the user’s average bedtime, *a*, *b*, and *c* are fitted quadratic parameters, *S* and *E* are fitted parameters for the start time and end time of the parabolic section of the model, and *d* is the constant portion of the model, which, in order to make the total sum to 1 is calculated as



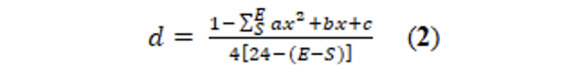



In model fitting, a series of fitting runs are performed, comprising a grid search for all valid combinations of *S* and *E* in 15-minute increments. Within each run, which is to say, for each tested combination of *S* and *E*, we use the R statistical software built-in polynomial fit function to find optimal values for the quadratic parameters for the quadratic portion of the model. Then *d* is calculated for that run using equation 2, and then all these parameters are fed into equation 1 to yield a set of 96 modeled *r* values for each run, corresponding to the expected frequency of Reddit posting of users among each of the 96 15-minute increments of the 24-hour day. Next, the modeled *r* values are compared with the empirical distribution of Reddit posting frequency in the training data, to yield a mean squared-error for the best-fitting model of that run. The final model is the model across the full sweep of *S* and *E* combinations that yields the lowest mean squared-error overall.

### Estimating Bedtimes

While the unified model has the advantage of readily incorporating information from users with any bedtime, the same property means that it does not encode any specific bedtime and is of no direct use in estimating another user’s bedtime. Therefore, from the unified model, we then generated a set of parallel versions of the model in which the moment of bedtime is relabeled with any local time, such as 9 PM, 9:15 PM, and so on (1 for each 15-minute interval of the 24-hour day) and all other points shifted accordingly (Figure 1D). Then, in the testing set, we tabulated each user’s circadian distribution of Reddit posting and determined whether a given user’s distribution most resembles (by Spearman correlation) the 9 PM-bedtime curve, the 3 AM-bedtime curve, and so on to estimate the user’s bedtime.

### k-Fold Cross-Validation

We next sought to test whether our chosen model is subject to overfitting using k-fold cross-validation. To ensure adequate sample size in training and hold-out sets, we set k=3. The 159 eligible users were each randomly assigned to one of 3 equally sized partitions. Within each of the 3 cross-validation runs, 1 partition was successively designated as the hold-out partition and the other 2 as the training partitions. The model described above was then trained on the subset of the 159 eligible users assigned to the 2 training partitions of that cross-validation run and tested on the respective hold-out partition. To ensure that results were not dependent on stochastic factors in assigning training and hold-out sets, we performed 10 full iterations of 3-fold cross-validation using 10 different random seeds to assign eligible users to partitions.

### Comparison With an Alternative Model

To benchmark our model against alternatives, we compared the performance of our model to a machine learning random forests approach. We used R’s ranger package version 0.14.1 to train a random forest model to estimate bedtimes from bins of Reddit posting rates across the 96 intervals of 15 minutes in the 24-hour day among users in the training data. The forest had 10,000 trees and otherwise default meta-parameters.

### Ethical Considerations

This study was determined exempt from IRB review by the Duke University Health System IRB with protocol numbers Pro00106817 and Pro00106782, and Duke Campus IRB Pro2022-0339. For the private survey, participants indicated their consent electronically; they were informed that we will not share their usernames with third parties. For the public data, the Reddit data usage agreement states that posting publicly to Reddit acts as consent to share this information publicly and freely. Newly data generated by this study (private surveys) are included as a data file with associated R package in an anonymous and deidentified fashion. Some public data used in this study that will be useful in downstream applications (eg, usernames of public data) can only be obtained from the authors of this study through a data use agreement to qualified researchers agreeing not to seek to identify the person behind the pseudoanonymous usernames and not to share the data with others. Subjects received no compensation for participating.

## Results

### Sample Characteristics

The final training and testing set consisted of 159 Reddit users, their self-reported bedtimes, time zones, and the time stamps of their collective 2,178,245 Reddit posts. Of the 159 users, 42 had been recruited privately and 117 were involved passively. Of these users, 82 (51.6%) were located in the United States, 21 (13.2%) in Canada, 17 (10.7%) in the United Kingdom, 23 (14.5%) elsewhere in Europe, and 16 (10.1%) spread in the rest of the world. The mean reported bedtime of the group was 11:47 PM.

The final application set consisted of 51,372 Reddit users, their time zones, and the time stamps of their collective 140,135,349 posts.

### Reddit Posting Declines After Self-reported Bedtime

Our overall goal was to develop a model for inferring the bedtimes of Reddit users from the time stamps of their posts. The core assumption of our approach is that bedtimes and time stamps are meaningfully related. Our first task was to test this assumption. Specifically, if bedtimes and time stamps are meaningfully related, we predicted that users would post less to Reddit for the night starting at around their bedtimes. To test this prediction, we analyzed the circadian distribution of the time stamps of the posts of the 159 users in the training set, stratified by their self-reported bedtime.

**Figure 2:**
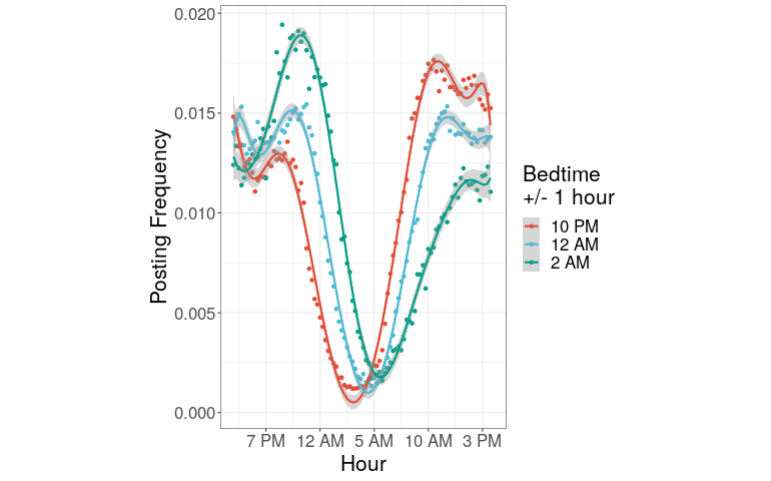
Circadian posting rhythms of Reddit. The x-axis lists the time of day, the y-axis gives the percent of all posts that are submitted at that time of day, stratified by bedtime. Bedtime groups: Red: 9 PM-10:45 PM; Blue: 11 PM-12:45 AM; and Green: 1 AM-2:45 AM.

We found that users post to Reddit much less frequently at night, in a manner strongly associated with their particular self-reported bedtime (Figure 2). Upon visual inspection of users with bedtimes ranging from 9 PM to 3 AM, the nighttime lull of users with earliest bedtimes (10 PM, ± 1 hour, in red) is most left-shifted, the lull for users with the latest bedtimes (2 AM, ± 1 hour, in green) is most right-shifted, and the lull for users with intermediate bedtimes (12 AM, ± 1 hour, in blue) is in the middle. These data indicate that the quiet nighttime hours of Reddit’s posters reflect in part those posters’ particular sleeping times and not merely a demand-side consequence of the sleep cycle of Reddit’s readers. The data also show that whatever automated posting passes our filters is not large enough to swamp the circadian rhythms of users who only post when awake.

**Figure 3:**
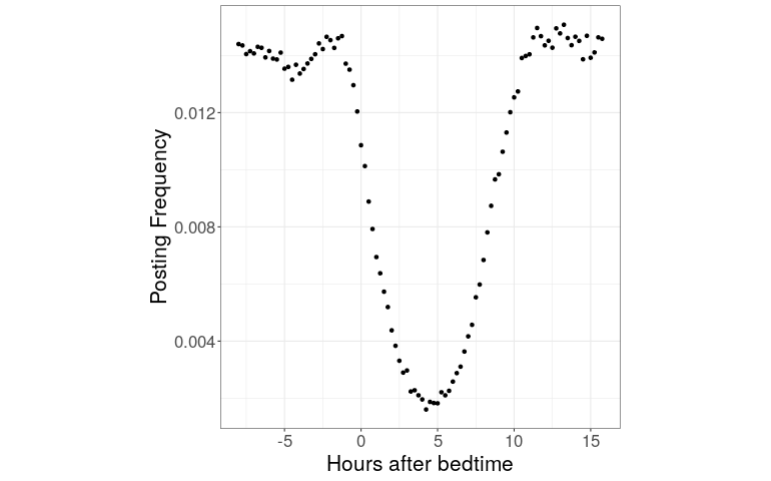
Aggregate circadian posting, relative to bedtime. Users with all bedtimes have been aggregated onto the same curve. The x-axis lists the time of day not by clock hours but by hours relative to bedtime.

The trend of Reddit posting declining after self-reported bedtime is more apparent when we combine the data from all labeled participants in a unified way. A comprehensive aggregate observed circadian profile is plotted in Figure 3. We were able to combine the Reddit posting frequencies by time bin for users with disparate bedtimes by indexing time bins by the number of hours after a user’s reported bedtime. The comprehensive aggregate observed circadian profile highlights the dominant trend of the users’ posting less to Reddit after their bedtime before resuming several hours later.

In this first section, we have demonstrated that our core assumption holds: bedtimes and Reddit time stamps are indeed interrelated. This provides a foundation for future sections. Moreover, the analysis here also serves as a quality control check that we have performed data integration correctly at a technical level.

### An Idealized Model of Circadian Posting

Having shown that bedtimes and time stamps are related, we next sought to develop a model that relates these quantitatively. For inspiration, we turned again to Figure 3, and noted, qualitatively, certain features in the overall shape of the posting frequency distributions: approximately constant posting frequency during the day, with an approximately parabolic depletion in posting that starts at or near the user’s bedtime.

**Figure 4:**
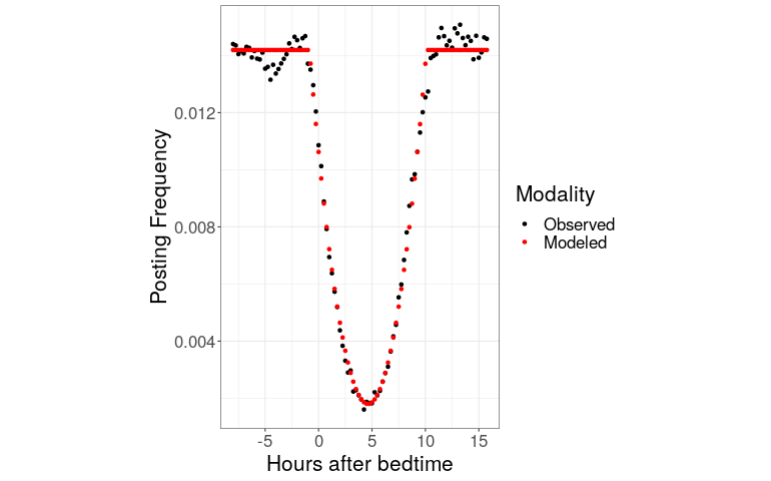
Modeled and observed Reddit posting frequencies by hour relative to bedtime. In black is the observed aggregated posting frequency of users by hour after bedtime, as in Figure 3. In red is the fitted model that is parabolic in the nighttime hours and flat during the daytime hours.

We wanted to see how good a fit a model with these properties would have for the observed data. Figure 4 shows our model plotted against aggregate data. The Pearson correlation between expected and mean observed posting frequencies was 0.996. In the best-fitting model, the values for the parameters in equations 1 and 2 (Methods) are as follows: *S* is 0.75, E is 10, *a*, *b*, and *c* are 4.12×10^–4^, 3.81×10^–3^, and 1.06×10^–2^, respectively, and *d* is 1.42×10^–2^. In the best tested model, Reddit posting begins to appreciably decline 45 minutes before a user’s bedtime, reaches a nadir 4.75 hours after bedtime that is 87% lower than the daytime posting rate, and returns to baseline 10.25 hours after bedtime. We found a remarkably simple and strong quantitative relationship between Reddit time stamps and bedtimes.

### Estimating Individual Bedtimes

Having demonstrated the fit of our model to aggregate data, we next sought to test whether our model could be used to predict the bedtimes of individual users. To predict bedtimes, we fit the idealized model to an individual user’s time stamp distribution and estimate the bedtime as 45 minutes prior to the start of the parabolic portion of the model.

**Figure 5:**
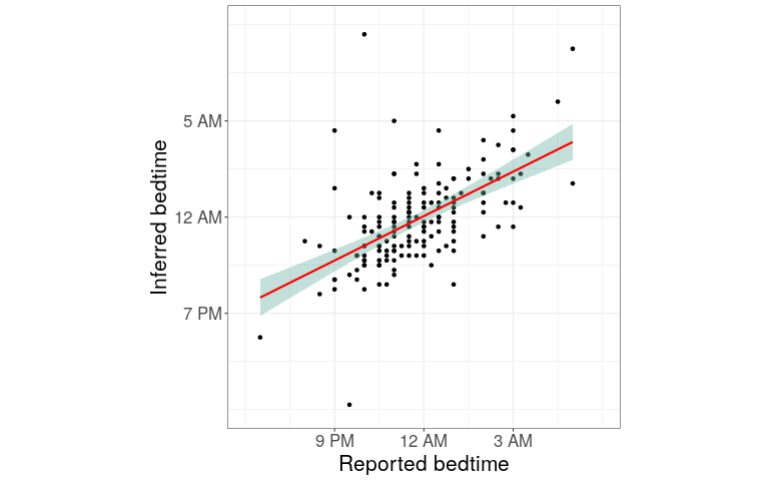
Estimated versus reported bedtimes for individual Reddit users. Each point represents one of the 159 Reddit users included in the training and testing data, his or her reported bedtimes (x-axis), and his or her estimated bedtime as inferred from the time stamps of the user’s posts (y-axis). The trend line is shown with its SE.

We quantified the accuracy of our model (Figure 5). The Pearson correlation coefficient in polar coordinates between inferred and reported bedtimes was 0.61 (*P*<.001). In 90 of 159 cases (56.6%), our estimate was within 1 hour of the reported bedtime. In 128 cases (80.5%), our estimate was within 2 hours of the reported bedtime. The residuals were centered on zero and approximately symmetric (Multimedia Appendix 2). The correlation we observed between estimated and reported bedtimes is comparable to the previously reported 0.47 correlation between self-reported and polysomnographically derived values for the related sleep parameter of sleep duration [24].

### Testing for Overfitting Using k-Fold Cross-Validation

To test the robustness of our model to overfitting, we employed k-fold cross-validation (Methods). Effectively, this procedure allowed all 159 labeled users to be used as validation data as part of 1 hold-out-set or another. The 10 runs of 3-fold cross-validation involved 30 validation tests of the model on held-out data. All 30 tests produced a statistically significant (*P*<.05) positive Pearson correlation between estimated and reported bedtimes. The median correlation of estimated and reported bedtimes among the training partitions was 0.61 (IQR 0.59-0.68). The median correlation among the hold-out partitions was 0.62 (IQR 0.57-0.66). The fact that accuracies in the hold-out partitions were comparable to accuracies in the training partitions indicates that the model is not appreciably subject to overfitting.

### Comparison With an Alternative Model

Random forests are a strong general purpose machine learning technique requiring minimal fine-tuning, making them an attractive comparison for our simple parametric model. We trained a random forest on the training data and computed a best-case accuracy as the correlation between the random forest’s predictions for the training data and the reported bedtimes for the training data. The random forest’s predictions correlated with the reported bedtimes of the training data with Pearson ρ of 0.47 (*P*<.001). This is less accurate than 0.61 correlation obtained with our chosen parametric model. Despite the simplicity of our model, our model has better performance than the popular machine learning technique of random forests, indicating that our model is well suited to our use case.

### Application to Users With Known Time Zones

**Figure 6:**
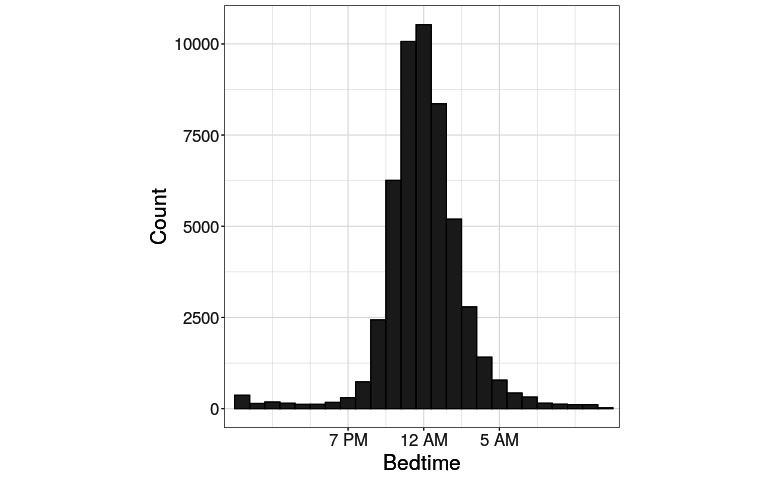
Estimated bedtime of 50,000 Reddit users. A histogram of inferred bedtimes for more than 50,000 Reddit users from the timestamps of their posts.

Having validated our model, we next sought to apply it to a wider user set to characterize the bedtimes of thousands of Reddit users. We used our model to estimate bedtimes for the 51,372 users identified in [21] as having publicly indicated their locations for whom we were able to download at least 250 time stamps. We estimated bedtimes in this set. A histogram of estimated bedtime is depicted in Figure 6. The mean bedtime was 12:12 AM (SD 2.41 hours). Among US-based users, the average estimated bedtime is 12:02 AM. This is 22 minutes later than the observed bedtimes in the nationally representative NHANES study [25]. Perhaps, this difference reflects Reddit's young, tech-savvy user base [26].

In additional analyses, we investigated how estimated bedtimes for these 50,000 users changed by day of week and by month and year (Multimedia Appendix 2).

## Discussion

### Principal Findings

There is a growing interest in studying the sleep patterns of social media users. Most prior work in this area has proceeded through the recruitment of subjects to self-report or electronically track sleep patterns and self-report social media usage. Some enterprising researchers have attempted to infer social media users’ bedtimes from the time stamps of their posts, which obviates the need for subject recruitment, avoids some aspects of selection and recall bias, and lends itself well to the integrated analysis of bedtimes with the contents of users’ posts. Linguistic approaches in the literature have been used to identify individuals reporting insomnia, but not to identify bedtimes; prior work to estimate specifically bedtimes from social media data did not use external data to verify their assumptions.

In this work, we developed and tested a model for inferring the bedtimes of Reddit users from the time stamps of their posts. Our accuracy in the testing data is comparable to the correlation of polysomnographically derived sleep duration with self-reported sleep durations [25].

### Limitations

One limitation of our approach is that it can only generate estimates for users who post frequently to Reddit (such as 250 or more lifetime posts), and these users may not be representative of Reddit users who mostly read Reddit without frequently posting. Likewise, Reddit itself is not representative of all social media users or the population at large, and for example, skews younger, more male, and more tech-savvy than Facebook or than the general population. Moreover, we have not validated the proposed method with other social media platforms, which might have a different balance of laptop versus mobile phone users that could influence the timing of their last post before sleep [27].

### Future Directions

There are a number of future directions for improving, extending, and applying this model. A more complex model might make use of adaptive width in the parabolic depletion. The framework could be readily extended to other sleep parameters such as wake times or sleep durations or to other social media platforms with fit-for-purpose training data. Researchers can apply our model to study the relationship between bedtimes on Reddit and any of the many features users share about their lives on Reddit.

### Conclusions

Our work enables researchers to study the relationship between bedtimes and any of the aspects of life users share on social media, in a rigorous, validated manner without the need to recruit a cohort.

## Appendix

Multimedia Appendix 1 Rules for coding free-text reported bedtimes of Reddit users.

Multimedia Appendix 2 Supplemental methods and results.

## Abbreviations

IRB: institutional review board
UTC: universal coordinated time

## Data Availability

Reddit data are publicly available from Baumgartner et al [22]. Our R package is publicly available in Meyerson [28]. We also plan to make available our precomputed estimates of the bedtimes of more than 50,000 Reddit users to qualified researchers who complete a data usage agreement.

## References

[1] Chattu VK, Manzar MD, Kumary S, Burman D, Spence DW, Pandi-Perumal SR (2018). The global problem of insufficient sleep and its serious public health implications. Healthcare.

[2] Kecklund G, Axelsson J (2016). Health consequences of shift work and insufficient sleep. BMJ.

[3] Owens J (2014). Insufficient sleep in adolescents and young adults: an update on causes and consequences. Pediatrics.

[4] Chaput JP, Dutil C, Featherstone R, Ross R, Giangregorio L, Saunders TJ, Janssen I, Poitras VJ, Kho ME, Ross-White A, Zankar S, Carrier J (2020). Sleep timing, sleep consistency, and health in adults: a systematic review. Appl Physiol Nutr Metab.

[5] Zerbini G, van der Vinne V, Otto LKM, Kantermann T, Krijnen WP, Roenneberg T (2017). Lower school performance in late chronotypes: underlying factors and mechanisms. Sci Rep.

[6] Norbury R (2021). Diurnal preference and depressive symptomatology: a meta-analysis. Sci Rep.

[7] Taylor BJ, Bowman MA, Brindle A, Hasler BP, Roecklein KA, Krafty RT, Matthews KA, Hall MH (2020). Evening chronotype, alcohol use disorder severity, and emotion regulation in college students. Chronobiol Int.

[8] Scott H, Woods HC (2019). Understanding links between social media use, sleep and mental health: recent progress and current challenges. Curr Sleep Med Rep.

[9] Scott H, Biello SM, Woods HC (2019). Social media use and adolescent sleep patterns: cross-sectional findings from the UK millennium cohort study. BMJ Open.

[10] Levenson JC, Shensa A, Sidani JE, Colditz JB, Primack BA (2017). Social media use before bed and sleep disturbance among young adults in the United States: a nationally representative study. Sleep.

[11] Hill DL (2020). Social media: anticipatory guidance. Pediatr Rev.

[12] Shimoga SV, Erlyana E, Rebello V (2019). Associations of social media use with physical activity and sleep adequacy among adolescents: cross-sectional survey. J Med Internet Res.

[13] Hamilton JL, Lee W (2021). Associations between social media, bedtime technology use rules, and daytime sleepiness among adolescents: cross-sectional findings from a nationally representative sample. JMIR Ment Health.

[14] Varghese NE, Santoro E, Lugo A, Madrid-Valero JJ, Ghislandi S, Torbica A, Gallus S (2021). The role of technology and social media use in sleep-onset difficulties among italian adolescents: cross-sectional study. J Med Internet Res.

[15] Kaur P, Dhir A, Alkhalifa A, Tandon A (2021). Social media platforms and sleep problems: a systematic literature review, synthesis and framework for future research. Internet Res.

[16] Talbot J, Charron V, Konkle AT (2021). Feeling the void: lack of support for isolation and sleep difficulties in pregnant women during the COVID-19 pandemic revealed by twitter data analysis. Int J Environ Res Public Health.

[17] McIver DJ, Hawkins JB, Chunara R, Chatterjee AK, Bhandari A, Fitzgerald TP, Jain SH, Brownstein JS (2015). Characterizing sleep issues using Twitter. J Med Internet Res.

[18] Sakib AS, Mukta MSH, Huda FR, Islam AKMN, Islam T, Ali ME (2021). Identifying insomnia from social media posts: psycholinguistic analyses of user tweets. J Med Internet Res.

[19] Yoshida M, Kojima T, Matsumoto K, Kita K (2021). Toward analyzing relations between sleeping time and social networking service texts?: prediction of the tweet time span using the last tweet of the day. Int J Adv Intell.

[20] Mieskes M, Fort K, Névéol A, Grouin C, Cohen KB (2019). NLP Community perspectives on replicability. https://hal.archives-ouvertes.fr/hal-02282794.

[21] Harrigian K (2018). Geocoding without geotags: a text-based approach for reddit. https://aclanthology.org/W18-6103.

[22] Baumgartner J, Zannettou S, Keegan B, Squire M, Blackburn J (2020). The pushshift reddit dataset. Proc Int AAAI Conf Web Soc Media.

[23] Orabi M, Mouheb D, Al Aghbari Z, Kamel I (2020). Detection of bots in social media: a systematic review. Inf Process Manag.

[24] Lauderdale DS, Knutson KL, Yan LJ, Liu K, Rathouz PJ (2008). Sleep duration: how well do self-reports reflect objective measures? the CARDIA sleep study. Epidemiology.

[25] Urbanek JK, Spira AP, Di J, Leroux A, Crainiceanu C, Zipunnikov V (2018). Epidemiology of objectively measured bedtime and chronotype in US adolescents and adults: NHANES 2003-2006. Chronobiol Int.

[26] Shatz I (2017). Fast, free, and targeted: reddit as a source for recruiting participants online. Soc Sci Comput Rev.

[27] Villanti AC, Johnson AL, Ilakkuvan V, Jacobs MA, Graham AL, Rath JM (2017). Social media use and access to digital technology in US young adults in 2016. J Med Internet Res.

[28] Meyerson W (2022). BEDDiT. Github Respository.

