# Supplementary material for: Estimation of Bedtimes of Reddit Users: Integrated Analysis of Time Stamps and Surveys
Source: JMIR Form Res. 2023 Jan 17;7:e38112. doi: 10.2196/38112 (PMC9890352; doi:10.2196/38112)
Supplement: Multimedia Appendix 1 [file formative_v7i1e38112_app1.docx]

1. **Simple**: If a user reports a single unambiguous 24-hour or 12-hour-with-explicit-meridian numerical bedtime or sleep time, and this time falls between 7 pm and 7 am, record as reported. If user reports rare exceptions, ignore these exceptions.

Example: “I go to bed at 3 am” -> 3:00 AM

Example: “I go to bed at midnight” -> 12:00 AM

Example: “I go to bed at 2300 -> 11:00 PM

Example: “I usually go to bed at 10 pm, but I stay up until midnight on New Year’s Eve” -> 10:00 pm

2. **Contextual**: If a user reports a single 12-hour numerical bedtime or sleep time with omitted meridian and without other clear context cues, record 8:00-11:59 as PM, 12:00-2:59 as AM, and all other times as NA.

Example: “I go to bed at 9” -> 9:00 PM

Example: “I go to bed at 4” -> NA

3. **Range:** If a user reports either only a bedtime or only a sleep time, and reports this time as a range, or as a split between two different times for two types of nights where both types of nights are common, if the range or split spans 3 hours or fewer record as the mean of the range or split, if the range and/or split spans more than 3 hours, record as NA. If a range is reported for each fork of a split, compute the average for each split, then the average between splits. Ignore rare types of night.

Example: “I go to bed anywhere from 11 pm to 1 am” -> 12:00 AM

Example: “On weekdays I go to bed at 10 pm, on weekends at 1 am” -> 11:30 PM

Example: “I go to bed anywhere from 8 pm to 11:30 pm” -> NA

Example: “In the schoolyear, I go to bed between 9 pm and 10 pm, in the summer I go to bed between 10 pm and 12 am” -> 10:15 pm [average of 9:30 and 11:00]

Example: “If I have a shift that evening I go to bed between 1 am and 2 am, otherwise between 10 pm and 11 pm” -> NA [since 10 pm to 2 am is more than 3 hours]

Example: “usually between 10 pm and 12 am, on occasion as late as 2 am” -> 11:00 PM [since 2 am is rare]

4. **Actual:** If a user reports both an intended sleep time and an actual sleep time, or reports both a bedtime and a sleep time, record as the actual sleep time. However, if mean intended and mean actual differ by more than 3 hours, report as NA.

Example: “I try to go to bed by 9:30 pm, but actually it’s more like 10:30 pm” -> 10:30 PM

Example: “I go to bed at 10:00 pm but I typically don’t fall asleep until 12:00 am” -> 12:00 AM

Example: “I go to bed at 8 pm but don’t fall asleep until 4:00 am” -> NA

5. **Bounded:** If a user reports their bedtime / sleep time as later (or no earlier) than X, record as X+30 min; if reports earlier (no later) than X, record as X-30 min.

Example: “I don’t usually go to bed until after midnight” -> 12:30 AM

Example: “I am usually in bed before midnight” -> 11:30 PM

Example: “I go to bed no later than 2 am” -> 1:30 AM

Example: “I go to bed no earlier than 3 am” -> 2:30 AM

6. **Unexpected and unexplained:** If a user reports a bedtime / sleep time after 7 am and before 7 pm, record as NA unless user acknowledged that their bedtime / sleep time is unusual, in which case record as reported.

Example: “8 am” -> NA

Example: “9 am, I work the night shift” -> 9:00 AM

7. **Temporary:** If a user indicates that their current bedtime/sleep time is temporary or a recent change, record as NA.

Example: “Lately I’ve been going to bed at 10 pm” -> NA

Example: “I’ve been staying up until 4 am now that I’m on winter break” -> NA

8. **Denial:** If a user denies sleeping, record as NA.

Example: “assuming I go to bed at all” -> NA

9. **Precise**: If a user reports an exact sleep minute that is not a multiple of 5, record a time between 8 pm and midnight as is, and record a time after midnight as NA unless the user explains the precision behind their report of their bedtime.

Example: “I go to bed at 9:33 pm” -> 9:33 PM

Example: “4:52 am” -> NA

Example: “according to my Fitbit, my average bedtime is 2:03 am” -> 2:03 AM

x. **Irrelevant of uninterpretable:** If a post does not meet any of the criteria above, and cannot be interpreted as a number by a reasonable reader, record as NA.

Example: “Nice man, I wish I could sleep that much” -> NA

Example: “I go to sleep quite late” -> NA

0. **Judgment required:** If a post does not meet any of the criteria above, but may be interpreted by a reasonable reader, leave blank and submit for adjudication.

Example: “Same” -> _____

Example: “I usually wake up at 5 and get 8 hours of sleep” -> _____
