# Supplementary material for: Estimation of Bedtimes of Reddit Users: Integrated Analysis of Time Stamps and Surveys
Source: JMIR Form Res. 2023 Jan 17;7:e38112. doi: 10.2196/38112 (PMC9890352; doi:10.2196/38112)
Supplement: Multimedia Appendix 2 [file formative_v7i1e38112_app2.docx]

# Supplemental Methods and Results for: “Estimation of Bedtimes of Reddit Users: Integrated Analysis of Timestamps and Surveys”

## Residuals when estimating individual bedtimes

To better investigate model fit, we examined the distribution of residuals of the model within the training data. As elsewhere in the manuscript, we used circular coordinates to calculate residuals to correct for distortions of clock time in Euclidean space; for instance, the correct difference between -11 hours with respect to midnight (1 PM) and +11 hours with respect to midnight (11 AM) is a difference of 2 hours and not the Euclidean 22 hours. The mean residual was 0.02 hours, indicating that the model does not have a meaningful systematic tendency to either underestimate or overestimate bedtimes. The median absolute residual was 1 hour, indicating that half of all bedtime estimations are within 1 hour of the self-reports. The mean absolute residual was 1.38 hours. Overall, the residuals are approximately centered on zero and symmetric (Supplemental Figure 1). These results indicate that the model is not making large systematic errors and provides an assurance of model fit.

### Supplemental Figure 1: Residual for estimating individual bedtimes

Supplemental Figure 1: Histogram of the difference between estimated bedtimes and reported bedtimes for 159 included Reddit users with reported bedtimes.

## Analysis of social jet lag

The core functionality of our model can be readily extended through pre-processing steps. For instance, we demonstrate how to breakdown estimated bedtimes by the day of week by splitting the timestamp data into separate sections by day of week, and then apply the model separately to each day-of-the-week slice. In this analysis we restricted analysis to the 30,421 users of the application set located in the US and Canada to simplify concerns related to weekends being practiced differently in different cultures. The average estimated bedtime across these users is 32 minutes later on Saturdays than on Mondays. An exception is that users with the latest average bedtimes have similar estimated bedtimes each day of the week (Supplemental Figure 2). These results reproduce prior studies which demonstrate a social jet lag as people adjust to the constraints of work vs their preferred rhythms [1].

### Supplemental Figure 2: Bedtime by day of week, stratified by week-wide average bedtime

Supplemental Figure 2: The x-axis gives the day of the week, the y-axis lists the average estimated bedtime for a group of Reddit users on that day of the week; Reddit users are grouped by their average week-wide estimated bedtime.

This pre-processing step is also useful for estimating user chronotypes. Given the constraints of school and work during the workweek, weekend bedtimes are often taken to be a better representation of a person’s chronotype than are workweek or average bedtimes. Thus, when we examine a very late chronotype with a preference of bedtimes between 2 AM and 4 AM, the default model estimates that 11.6% of US and Canadian users of the application set meet this definition, but if we restrict attention to weekend bedtimes, using the pre-processing technique above, then the same figure is 16.0%.

## Impact of COVID-19 lockdowns on estimated bedtimes

We next sought to test whether the COVID-19 pandemic was associated with changes in Reddit users’ bedtimes. To accomplish this, we applied the model separately to each user for each month and year that was well-represented in the Reddit data, focusing on the recent years of 2015 to 2021, and then calculated the average bedtime across users for each month and year. We observe that bedtimes are significantly later starting in March 2020 when the pandemic hit. Specifically, a linear-smoothed estimated for users’ bedtime in March 2020 is 18 minutes later than what would have been predicted based on the pre-pandemic trend, but then restores to the previous trend (Supplemental Figure 3). These results likely reflect the disruption in school and work caused by the COVID-19 pandemic lockdowns and changing bedtime habits during this period [2]. We also notice a pattern in which for several years before the pandemic, estimated bedtimes creep slightly earlier with time, perhaps reflecting the effect of aging cohorts [3]. On the first author’s GitHub, we provide sample code for interested researchers to incorporate the effects of year and month on bedtime in their analyses.

### Supplemental Figure 3: Estimated bedtimes by year and month straddling COVID-19 lockdowns

Supplemental Figure 3: Each point represents the estimated bedtime (y-axis) for 50,000 Reddit users at a given month and year (x-axis). Points have been color-coded to indicate whether they occurred before (blue) or after (red) the start of the COVID-19 lockdowns, together with their trend lines and the standard error of those trend lines.

# Supplemental References

1. Wittmann M, Dinich J, Merrow M, Roenneberg T. Social Jetlag: Misalignment of Biological and Social Time. Chronobiol Int [Internet]. 2006 Jan 1 [cited 2022 Nov 21];23(1–2):497–509. Available from: https://doi.org/10.1080/07420520500545979

2. Leone MJ, Sigman M, Golombek DA. Effects of lockdown on human sleep and chronotype during the COVID-19 pandemic. Curr Biol [Internet]. 2020 Aug 17 [cited 2022 Nov 21];30(16):R930–1. Available from: https://www.sciencedirect.com/science/article/pii/S0960982220310071

3. Thomas SJ, Lichstein KL, Taylor DJ, Riedel BW, Bush AJ. Epidemiology of bedtime, arising time, and time in bed: analysis of age, gender, and ethnicity. Behav Sleep Med. 2014;12(3):169–82.
